# Long Non-Coding RNAs in Cardiac Hypertrophy

**DOI:** 10.3389/fmmed.2022.836418

**Published:** 2022-03-08

**Authors:** Nicolò Mangraviti, Leon J. De Windt

**Affiliations:** Department of Molecular Genetics, Faculty of Science and Engineering, Faculty of Health, Medicine and Life Sciences, Maastricht University, Maastricht, Netherlands

**Keywords:** lncRNA, circRNAs, cardiac hypertrophy, heart, heart disease

## Abstract

Heart disease represents one of the main challenges in modern medicine with insufficient treatment options. Whole genome sequencing allowed for the discovery of several classes of non-coding RNA (ncRNA) and widened our understanding of disease regulatory circuits. The intrinsic ability of long ncRNAs (lncRNAs) and circular RNAs (circRNAs) to regulate gene expression by a plethora of mechanisms make them candidates for conceptually new treatment options. However, important questions remain to be addressed before we can fully exploit the therapeutic potential of these molecules. Increasing our knowledge of their mechanisms of action and refining the approaches for modulating lncRNAs expression are just a few of the challenges we face. The accurate identification of novel lncRNAs is hampered by their relatively poor cross-species sequence conservation and their low and context-dependent expression pattern. Nevertheless, progress has been made in their annotation in recent years, while a few experimental studies have confirmed the value of lncRNAs as new mechanisms in the development of cardiac hypertrophy and other cardiovascular diseases. Here, we explore cardiac lncRNA biology and the evidence that this class of molecules has therapeutic benefit to treat cardiac hypertrophy.

## Introduction

Heart failure (HF) is a highly prevalent disease and a leading cause of hospitalization and death that affects 23 million patients worldwide (Ziaeian and Fonarow, 2016). From a clinical point of view, HF is defined as the state of deterioration of the heart where it can no longer supply sufficient blood to meet the circulatory demands of the organism. Heart transplantations are still the only genuine curative interventions for patients with advanced forms of HF as contemporary pharmacotherapy is largely palliative and merely aimed to slow the progression of the disease (Ziaeian and Fonarow, 2016). The disease is typically preceded by structural remodeling of the heart where heart muscle cells undergo maladaptive growth without an increase in cell number in response to sustained stress or injury such as pressure- or volume overload to temporally sustain cardiac output, resulting in a measurable thickening of heart muscle walls. However, cardiac hypertrophy is also accompanied by a plethora of biochemical, molecular, metabolic and extracellular changes that provoke a decrease of pump function over time rather than preserving it, resulting in overt heart failure and a propensity for the occurrence of lethal arrhythmias (Dirkx et al., 2013). Accordingly, a better understanding of the molecular underpinnings of cardiac hypertrophy will help to clarify the maladaptive nature of this disease and may open new therapeutic targets for future treatment of hypertrophic heart diseases to reduce the number of HF patients.

**FIGURE 1:**
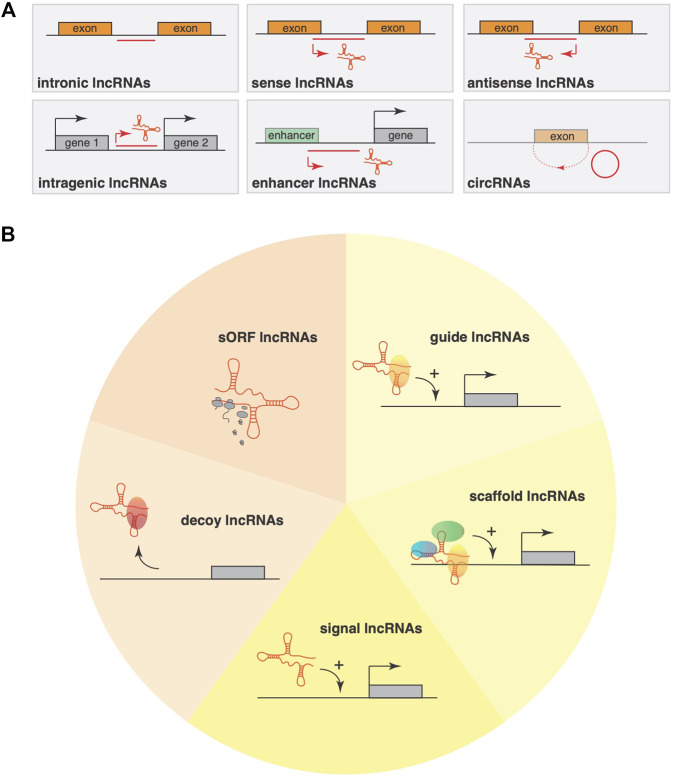
Classification systems for lncRNAs **(A)** LncRNA classification using purely their genomic location as input information allows a classification into six main categories: sense, antisense, bidirectional, intronic, intergenic and circRNAs. This classification mainly considers the position and orientation compared to the nearest gene or genomic features (promoters, enhancers). **(B)** An alternative classification system takes lncRNA mode of action into account, where lncRNAs have the ability to interact with DNA, other RNAs, with proteins or perform their function by pervasive transcription. Based on structural motifs within lncRNAs, an alternative classification divides lncRNAs into five classes, including short open reading frame (sORF) containing sORF lncRNAs that often encode functional, small peptide sequences; decoy lncRNAs that can either be located cytoplasmatic or nuclear and interact physically with RNAs or transcription factors to block their interaction with their cofactors or inhibit their function; signal lncRNAs that regulate gene expression in a time- and space dependent manner in response to cellular stimuli; guide lncRNAs that are predominantly nuclear and recruit chromatin-modifying complexes to specific loci either near the gene encoding the lncRNA; scaffold lncRNAs that can be either in the nucleus or cytoplasm and facilitate the assembling of multiple proteins, affecting histone modifications in the nucleus or that facilitate the assembling of ribonucleoproteins.

The Encyclopedia of DNA Elements (ENCODE) and the Functional Annotation Of Mouse (FANTOM) consortiums reveal that there are much more transcripts than originally predicted. Over 80% of the genome is transcribed in various classes of RNA and, surprisingly, coding transcripts account for just up to 3% of the genome, while the vast majority of other transcripts have no coding ability (Figure 1) (Consortium, 2012). For a long time, the presence of a large number of noncoding transcripts was dismissed as evidence for junk DNA or transcriptional noise, but more recent research reveals that a substantial proportion of these noncoding transcripts are functionally active RNA molecules that can be subdivided into small noncoding RNAs (<200 nt), such as microRNAs (miRs), transfer RNAs, and small nucleolar RNAs, on the one hand, and longer noncoding RNAs (>200 nt) that include ribosomal RNAs, natural antisense transcripts and other long noncoding RNAs (lncRNAs) (Engreitz et al., 2016; Quinn and Chang, 2016; O'Brien et al., 2018). While our knowledge of lncRNAs is still in its infancy, here we will summarize examples of lncRNAs that are involved in cardiac hypertrophy as it may provide useful insights how lncRNAs are functionally involved in the heart.

## Classification Systems of Lncrnas

LncRNAs are often polyadenylated and often devoid of evident open reading frames (ORFs). The human genome is estimated to contain 16,000 lncRNA genes (Sun et al., 2018), mostly transcribed by RNA polymerase II and only a small fraction experimentally investigated (Marchese et al., 2017). Unfortunately, studying lncRNAs represents a challenge for molecular biology as they are evolutionarily poorly conserved, show a relatively low expression level that—interestingly - is more restricted in a tissue- and time-specific manner, and mechanisms of action that remain incompletely understood (Bhat et al., 2016).

Unlike other classes of RNA, a universally accepted classification system is lacking for lncRNAs. Using purely the genomic location as input information allows a classification into five main categories: sense, antisense, bidirectional, intronic, and intergenic. This classification mainly considers the position and orientation compared to the nearest gene or genomic features (promoters, enhancers) (Cabili et al., 2011). Similarly, based on genomic position and features, alternative classifications have also been proposed. LncRNAs that are encoded at the promoter region or originate near the transcription start site (uaRNAs) represent up to 60% of all lncRNAs (Sigova et al., 2013), while those encoded from enhancer regions (eRNAs) amount to ∼20% (Kim et al., 2010). The remainder derive from coding genes or gene bodies can be subdivided into sense (gsRNAs) or antisense (gaRNAs) and amount to ∼5% of all lncRNAs (Modarresi et al., 2012), while those encoded in intragenic spaces are referred to as iRNAs or lincRNAs (Guttman et al., 2009). For instance, another widely used system is based on the cellular localization of lncRNAs, where they are subdivided into cytoplasmatic- or nuclear lncRNAs. Unfortunately, this system is not fully reliable either because only 30% of lncRNAs are found exclusively in the nucleus, 15% are found exclusively in the cytoplasm, while the remainder ∼50% show both nuclear and cytoplasmic localization (Kwon, 2013). To make matters worse, some lncRNAs can translocate between the nucleus and cytoplasm after stress or stimulation (van Heesch et al., 2014).

In terms of mode of action, lncRNAs can interact with other RNAs (e.g., mRNAs, miRNAs, circRNAs, and rRNAs), with proteins (mainly but not exclusively transcriptional factors and chromatin-remodeling complexes) or perform their function by pervasive transcription (Fatica and Bozzoni, 2014). One characteristic of lncRNAs is their ability to interact with proteins and is determined by their architecture and three dimensional folding. LncRNAs are single-strand RNAs that will try to reach a folded state with the lowest or most stable energy state. The form it takes will be determined from the primary sequence but also from its length, the electrical charges and interaction with molecular chaperones (Novikova et al., 2013). Indeed, the folding and 3D shape seems an evolutionary conserved characteristic since lncRNAs are less evolutionary conserved on the primary sequence, but more often maintain their 3D structure throughout species (Lai et al., 2018). Specific RNA motifs have been shown to interact with proteins, allowing lncRNA to sequester or interact with various proteins simultaneously. Finally, by being RNA molecules, lncRNAs can create RNA:RNA duplex which allow them to interact with other RNA classes (e.g., microRNAs) or in other circumstances they can form RNA:DNA duplexes (Trembinski et al., 2020), which allow them to interact to specific target sequences on the DNA.

Based on these molecular specifics, an alternative and comprehensive classification divides lncRNAs into four classes. The first class is represented as decoy lncRNAs that can either be located cytoplasmatic or nuclear and interact physically with transcription factors to block their interaction with their cofactors or inhibit their function (Wang and Chang, 2011). LncRNA *ROR* is an example of a decoy lncRNA, which activates the TESC promoter by repelling the histone G9A methyltransferase (Fan et al., 2015). Additionally, decoy lncRNAs can also sequester microRNAs such as *linc-MD1* that sponges miR-133 and miR-135 to regulate the expression of MAML1 and MEF2C, two transcription factors that activate muscle-specific gene expression (Cesana et al., 2011).

Signal lncRNAs can also be localized either in the cytoplasm or nucleus and regulate gene expression in a time- and space dependent manner in response to cellular stimuli and often are only expressed at specific times during development (Fatica and Bozzoni, 2014). Their task is to modulate gene expression of key genes by reshaping the chromatin in a specific locus as, for instance, *KCNQ1OT1* is expressed only during early development where it interacts with chromatin-modifying enzymes to trigger lineage-specific transcriptional profiles (Pandey et al., 2008).

Guide lncRNAs are predominantly nuclear and can recruit chromatin-modifying complexes to specific loci either near the gene encoding the lncRNA (*cis*-action), or distant target genes from the production site of the lncRNA (*trans*-action). *HOTAIR* is an example of a guide lncRNA that functions in trans to direct the chromatin modifier Polycomb Repressive Complex 2 (PRC2) to the developmental HOXD locus (Rinn et al., 2007). Instead, *HOTTIP* is a guide lncRNA encoded in the HOXA locus that binds WDR5 and the histone methyltransferase protein MLL and directs the WDR5/MLL complex towards activation of the HOXA locus (Wang et al., 2011).

Finally, scaffold lncRNAs can be either in the nucleus or cytoplasm and facilitate the assembling of multiple proteins, which may act on chromatin, affecting histone modifications in the nucleus or facilitate the assembling of ribonucleoproteins. Telomerase RNA *TERRA* is a classic example of an RNA scaffold that assembles the telomerase complex to maintain the ends of telomeres (Oliva-Rico and Herrera, 2017).

Even this classification system is not always adequate to unequivocally differentiate classes of lncRNA as some can simultaneously belong to multiple classes. For example, lncRNA *KCNQ1OT1* functions both as a scaffold and guide lncRNA at the same time. For this reason, some authors have decreased the above classification system to only three: guides, decoy and a combined class called a dynamic scaffold. And yet, even with this adaptation, not all lncRNAs would still fall in any category. Some lncRNAs for instance can influence proximal genes (in cis) just by the effect of their own transcription in a process called transcriptional interference. Indeed, *Airn* can modulate the Igf2r promoter as long as only a small fraction of *Airn* is transcribed as this lncRNA is antisense to Igf2r and its transcription influences the state of chromatin in the surrounding area with an inability of RNA Polymerase II to interact freely with the promoter of Igf2r (Latos et al., 2012).

Finally, although it is not often included in lncRNA classification systems, several studies revealed that a proportion of lncRNAs contain short open reading frames (sORFs) that encode for small proteins or micropeptides with largely overlooked but fundamental biological importance (Galindo et al., 2007; Kondo et al., 2010; Andrews and Rothnagel, 2014; Bazzini et al., 2014; Pauli et al., 2014; Anderson et al., 2015; Anderson D. M. et al., 2016; Nelson et al., 2016). For example, *Myoregulin* (MLN) is a micropeptide encoded by *LINC00948,* an important regulator of skeletal muscle physiology. This micropeptide peptide controls calcium re-uptake in the sarcoplasmic reticulum (SR) by Serca2a inhibition (Anderson et al., 2015). Recently, van Heesch and colleagues (van Heesch et al., 2019) used ribosome profiling to capture ribosomal footprints on human cardiac transcripts and inferred actively translated sORFs that encode previously unknown microproteins in over 169 lncRNAs and 40 circular RNAs that are expressed in the heart. Dozens of microproteins are expressed even from previously non-coding roles for lncRNAs, such as *DANCR* (also known as *ANCR* (Kretz et al., 2012)), *TUG1* (Young et al., 2005), *JPX* (Tian et al., 2010), *Myheart* (Han et al., 2014), and *UPPERHAND* (Anderson K. M. et al., 2016). This finding suggests that a number of translated lncRNAs that could be separately classified as sORF lncRNAs, likely have dual coding and noncoding roles and this duality should be considered when deciphering the function of this class of “non-coding” transcripts.

Next-generation sequencing (NGS) techniques such as deep RNA-seq or CAGE-seq and 3P-seq have greatly aided to provide genome-wide identification of relatively low abundantly expressed transcripts such as lncRNAs (Oliva-Rico and Herrera, 2017). The results of these profiling experiments are now incorporated in databases with collections of hundreds of lncRNAs with possible differential expression in pathological conditions in a variety of species (Table 1) (Latos et al., 2012).

**TABLE 1:** List of Open-Source databases with information on lncRNAs.

Database	Species	lncRNAs reported	References
LNCipedia	human	118.777	Volders et al. (2013)
NONCODE	16 species including human	487.164	Zhao et al. (2016)
LncRNADisease v2	4 species including human	19.166	Bao et al. (2019)
ChIPBase v2	10 species including human	10.200	Zhou et al. (2016)
deepBase	14 species including human	191.547	Zheng et al. (2016)
GENCODE	human, mouse	42.302	Frankish et al. (2019)
LincSNP	human	244.545	Ning et al. (2016)
LncRNAdb v2.0	human	295	Quek et al. (2015)
FANTOM5	human	19.175	Hon et al. (2017)
HDncRNA	Human, mouse, rat	2.304	Wang W. J. et al. (2018)

## Prohypertrophic Cardiac Lncrnas

***H19*** is a lncRNA that was first associated with genomic imprinting of the H19/Igf2 locus (Kaffer et al., 2001; Table 2). Subsequent studied demonstrated that *H19* is upregulated in pathological forms of cardiac hypertrophy and heart failure. Interestingly, *H19* also encodes for the small ncRNA miR-675 that is embedded in the *H19* locus and that can target Ca2+/calmodulin-dependent protein kinase IIδ (CaMKIIδ), a powerful inducer of cardiac hypertrophy (Liu et al., 2016). CTBP1 Antisense RNA 2 (*CTBP1-AS2*) is a novel lncRNA that can trigger the hypertrophic response as it is upregulated in hearts that undergo hypertrophy after transverse aortic constriction (TAC) surgery in mice, while, conversely, silencing of *CTBP1-AS2* attenuates hypertrophy in AngII agonist stimulation of cardiomyocytes in culture. Mechanistically, this lncRNA can stabilize the mRNA encoding Toll-like receptor four by recruiting the RNA-binding protein FUS/TLS, thereby triggering cardiac inflammation, a commonly observed phenomenon associated with cardiac hypertrophy (Luo et al., 2019). Cardiac Hypertrophy-Related Factor **(*CHRF*)** is upregulated in both hypertrophic mouse hearts and human biopsies of patients with heart failure and functions as a sponge for miR-489, regulating Myeloid differentiation primary response gene 88 (Myd88) as downstream target of miR-489. Myd88, a signal transduction adaptor involved in immune modulation, was demonstrated to suppress cardiomyocyte hypertrophy. Other studies have shown that mir-489 is not the only target of *CHRF* (Wang et al., 2014), as miR-93 can also bind CHRF and influence AKT3 activation status (Wo et al., 2018). LncRNA ***ROR*** acts as a decoy lncRNA that can trap miR-133, thereby influencing RhoA and Cdc42 downstream of Gαq/Gα11 signaling (Jiang et al., 2016). Cardiac Hypertrophy-Associated Transcript (*CHAST*) is a signal lncRNA that show temporal regulation of expression in cardiac hypertrophy in mice and silencing of *Chast* with a GapmeR *in vivo* attenuated the hypertrophic response. Conversely, *Chast* overexpression triggers cardiomyocyte hypertrophy. Further studies revealed that *Chast* negatively regulates Plekhm1, a multivalent endocytic adaptor involved in controlling selective and nonselective autophagy pathways resulting in adverse cardiac remodeling (Viereck et al., 2016).

**TABLE 2:** lncRNAs with pro-hypertrophic functions.

Name	Target	Function	Cell type	References
*H19*	miR-675	Regulation of CaMKIIδ	CM	Liu et al. (2016)
*MEG3*	miR-361-5p	Regulation of STAT3	CM/CF	Piccoli et al. (2017), Wu et al. (2018)
*Lnc-ROR*	mir-133	Regulation of RhoA and Cdc42	CM	Jiang et al. (2016)
*CHAST*	Plekhm1	Regulation of Autophagy	CM	Viereck et al. (2016)
*CAS15*	miR-432-5p	Regulation of TLR4	CM	Li et al. (2018)
*MIAT*	miR-150	Regulation of CaMKIIδ	CF	Wu et al. (2018)
*CTBP1-AS2*	FUS/TLS	Stabilize mRNA of TLR4	CM	Luo et al. (2019)
*SYNE1-AS1*	miR-525-5p	Regulation of SP1	CM	Zhu et al. (2016), Wang et al. (2019)
*Chear*	PCR2	Chromatin remodeling	CM/VM	Wang Z et al. (2016)

CM, Cardiomyocytes; CF, cardiac fibroblast; VM, ventricular myocytes.

## Anti-Hypertrophic Cardiac Lncrnas

Myosin Heavy Chain Associated RNA Transcripts (***MHRT***) was first identified as the antisense transcript of Myosin heavy chain 7 (Myh7). *MHRT* is highly expressed in the mouse and human adult heart and *MHRT* lentiviral overexpression reduced hypertrophic stress markers (Table 3). RNA immunoprecipitation (RIP) revealed that *MHRT* interacts with Brahma-related gene 1 (Brg1) to remodel chromatin and regulates gene expression such as *Myh6* (Han et al., 2014) and can influence the acetylation of the Myocardin protein in an HDAC5-dependent fashion (Miano, 2015; Luo et al., 2018). LncRNA ***TINCR*** is downregulated in hypertrophic hearts and lentiviral TINCR overexpression attenuates cardiac hypertrophy by interacting with EZH2, a functional enzymatic component of the Polycomb Repressive Complex 2 (PRC2). Chromatin-immunoprecipitation (ChIP) pull-down assays against EZH2 demonstrated that the *TINCR*-EZH2 complex binds directly to the CaMKII promoter and induces H3K27me3 histone modification (Shao et al., 2017). ***Lnc-Plscr4*** is a lncRNA is significantly increased in hearts from mice that underwent pressure overload-induced cardiac hypertrophy, where it reduces the expression and activity of miR-214, a prohypertrophic microRNA (Aurora et al., 2012; Azzouzi et al., 2013; Lv et al., 2018). The responsible downstream target of miR-214 is Mitofusin 2 (Mfn2), a protein that regulates mitochondrial fusion (Chen et al., 2011). Accordingly, reduced expression of Mfn2 attenuates protein synthesis in cardiac hypertrophy (Yu et al., 2018). *HOTAIR* is a lncRNA that has been extensively studied in embryonic development and cancer, but recently it was observed that it is substantially decreased in cardiac hypertrophy. Overexpression of *HOTAIR* suppresses angiotensin II-stimulated cardiomyocyte hypertrophy and following pressure overload surgery in mice. Mechanistically, *HOTAIR* was proposed to act as a ceRNA for miR-19, where miR-19 regulates Phosphatase and tensin homolog (PTEN) expression and indirectly regulates hypertrophy by influencing the activation status of PI3K/phospho-Akt signaling cascade in cardiomyocytes (Lai et al., 2017; Zhang et al., 2020).

**TABLE 3:** lncRNAs with anti-hypertrophic functions.

Name	Target	Function	Cell type	References
*Mhrt*	Bgr1	Chromatin remodeling	CM	Han et al. (2014)
*MAGI1-IT1*	miR-302e	Regulation of HDAC9	CM	Zhang et al. (2020)
*Plscr4*	miR-214	Regulator of Mfn2	CM	Lv et al. (2018)
*HOTAIR*	miR-19	Regulation of PTEN	CM/CF	Lai et al. (2017)
*XIST*	miR-101/miR-330–3p/miR-130	Regulator of S100B	CM	Xiao et al. (2019)
*TINCR*	EZH2	Chromatin remodeling	CM	Shao et al. (2017)
*CYTOR*	miR-155	Regulator of IKBKE	CM	Yuan et al. (2019)

CM, Cardiomyocytes; CF, cardiac fibroblast.

## CircRNAs

Circular RNAs are a class of non-coding RNAs with a continuous closed loop where the 3 and 5′ ends of exons of protein-coding genes or lncRNA genes are joined through back-splicing (Pamudurti et al., 2017; Table 4). CircRNAs are predominantly found in the cytoplasm and are highly stable. Moreover, they are evolutionarily conserved across the eukaryotic (Wang L et al., 2018). Based on their origin, circRNAs can be divided into four main classes: exonic circRNAs (ecircRNA), produced with the removal of each intron and generated from the back-spliced exons, while in exon-intron circRNAs (EIciRNA) the black-splicing takes place by including the intron inside the mature circRNA. The circularization of only introns produces circular intron RNAs (ciRNAs), while intergenic circRNAs are produced when two fragments from different genomic regions called intergenic circRNA fragments or ICFs are merged together and circularized (Wang F et al., 2016). CircRNAs can interact with proteins, or they can serve as decoys for microRNAs. For example, ***Circ-Foxo3*** inhibits proliferation by direct interaction with p21 and CDK2 and subsequent interference of the cell cycle (Du et al., 2016). ***CircACTA2*** instead acts as a microRNAs sponge in vascular smooth muscle cell where it interacts with miR-548f-5p, which in turn modulates the expression of smooth muscle *α*-actin (α-SMA) (Sun et al., 2017).

**TABLE 4:** CircRNA involved in cardiac hypertrophy.

Name	Target	Function	Cell type	References
*Circ-Foxo3*	p21 and Cdk2	Inhibit proliferation	CM/CF	Du et al. (2016)
*Circ-ACTA2*	miR-548f-5p	Regulation of *α*-SMA	VSMC	Sun et al. (2017)
*Circ-ZNF609*		Produce a micropeptide	CM	Legnini et al. (2017)
*HRCR*	miR-223	Regulation of ARC	CM	Wang K et al. (2016)
*CircSlc8a1*	miR-133	Regulation of SRF	CM	Lim et al. (2019)
*CircRNA_000,203*	miR-26b-5p and miR-140–3p	Regulation of Gata4	CM	Li et al. (2020)
*Circ-wwp1*	miR-23a	Unknown	CM	Yang et al. (2020)
*CircTmem56*	Unknown	biomarker	Plasma	Sonnenschein et al. (2019)
*CircDNAJc6*	Unknown	biomarker	Plasma	Sonnenschein et al. (2019)

CM, Cardiomyocytes; CF, cardiac fibroblast; VSMC, vascular smooth muscle cell.

Similarly, ***circRNA_000203*** can modulate the expression in Gata4 by binding microRNA miR-26b-5p and miR-140-3p (Li et al., 2020). Occasionally, circRNAs can encode for micropeptides by sORFs, an example is ***Circ-ZNF609*** that controls myoblast proliferation the production of a new small peptide generated by the back-splicing event (Legnini et al., 2017). Heart-related circRNA (***HRCR***) is a circRNAs that regulates cardiac homeostasis by modulating the expression of miR-223 *in vivo*, a well-known microRNA that induces cardiac hypertrophy (Wang K et al., 2016). Conversely, ***CircSlc8a1*** is highly expressed in heart failure, and can bind miR-133 to induce cardiac hypertrophy (Carè et al., 2007; Wang K et al., 2016). On the other hand, cirRNAs can act as early biomarkers of cardiac hypertrophy, *circTMEM56* and *circDNAJC6* could serve as indicators of disease severity in patients with hypertrophic obstructive cardiomyopathy (Sonnenschein et al., 2019)

## Experimental and Therapeutic Considerations

**FIGURE 2:**
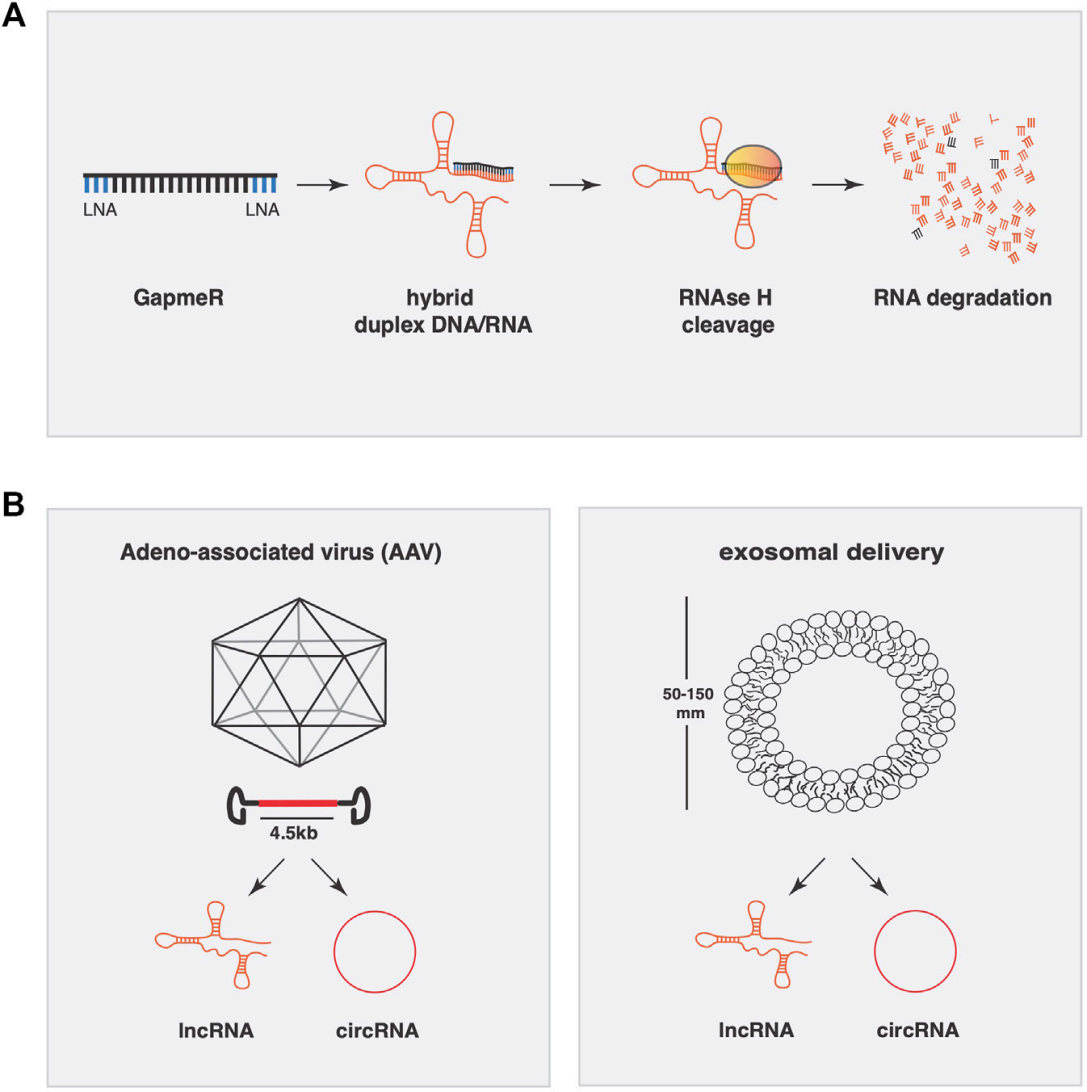
Conceptual approaches to manipulate lncRNAs expression for therapeutic applications. **(A)** Gapmer oligonucleotides have two LNA-modified linkers at the 3′ and 5′ end of the molecule to resist exo-and endonucleases once introduced into the cell or inside the extracellular space. Once bound to the lncRNA by full base pair complementarity, the dsRNA complex is degraded in an RNAse H dependent manner. **(B)** Adeno-associated viruses (AAV) have a packaging limit of 4.5 kb and can be engineered to transcribe lncRNAs or circRNAs. Exosomes are a specialized subgroup of extracellular vesicles ranging from 50–150 nm capable of carrying a variable composition of RNAs (lncRNA, microRNA, and circRNA).

One widely employed strategy to silence endogenous lncRNAs involves the introduction of double or single stranded antisense oligonucleotides in the form of siRNAs or antisense locked nucleic acid (LNA) containing oligos (GapmeRs), respectively. For short hairpins RNA or siRNAs, it is observed that those molecules seem more suitable for cytoplasmic lncRNAs rather than nuclear lncRNAs (Berezhna et al., 2006), although this might depend on various other parameters in siRNA design as well. GapmeRs are very potent antisense oligonucleotides with two LNA-modified linkers at 3′ and 5’ end of the molecule to resist exo-and endonucleases once introduced in the cell or in the extracellular space. Once bound with the lncRNA by full base pair complementarity, the dsRNA complex is degraded in an RNAse H dependent manner (Vickers et al., 2003). These molecules are highly efficient in silencing both cytoplasmatic and nuclear lncRNAs (Figure 2).

To achieve the opposite, i.e. supplementing or overexpressing a lncRNA, viral-mediated gene delivery remains the most reliable system (Figure 2). Viral vectors enhance cardiac delivery of polyanions, such as RNA, across cell membranes. One oftenly used viral vector is the non-pathogenic human parvovirus adeno-associated virus (AAV) for its genomic simplicity, possibility of generating high-titre vector preparations, and their capacity to deliver genes into postmitotic cells. The specific tropism of AAV serotypes drives their selectively for different tissues *in vivo*, where AAV9 stands as the most cardiotropic serotype in gene transfer studies in rodents. A limitation of AAV vectors is their packaging limit (∼4.5 kb), which may be exceeded by certain lncRNAs. Another viral vector is based on human adenovirus (HAdV). The best studied member of the HAdV species is serotype 5 (HAdV-5). HAdV-5 infects many cell types, including low-replicative or quiescent cell populations such as cardiomyocytes. The HAdV-5 genome is easy to engineer with large foreign DNA cloning capacity and can be produced on an industrial scale. All these attributes make HAdV-5 vectors the most preferred vector type used to date in vaccine, cancer, and gene therapy trials (Appaiahgari and Vrati, 2015).

Other systems of delivery are engineered nanoparticles and extracellular vesicles such as exosomes (Figure 2). Nanoparticles have demonstrated their potential in the oncological field as delivery systems for microRNAs, very efficiently and in general with relatively low toxicity. Furthermore, effective cardiac-specific nanoparticle delivery systems for *in vivo* use remain to be developed. In contrast, exosomes are a specialized subgroup of extracellular vesicles ranging from 50–150 nm and released from cells to the extracellular microenvironment where they can be found in various extracellular fluids including the systemic circulation. Naturally occurring exosomes carry a variable composition of proteins, lipids, RNA (mRNA, lncRNA, microRNA, and circRNA), and DNA, where they influence cell migration, angiogenesis, the immune response and tumor cell growth (Boriachek et al., 2018). Engineered extracellular vesicles could be used as engineered vehicles to temporally deliver lncRNAs. Proof of principle exist in cancer research, where exosomes that carry different lncRNAs to influence tumor development. *MALAT-1*, *BCAR4*, and *LncRNA-p21* are LncRNAs found in exosomes that traveled in the bloodstream derived from different types of cancers. Previous work has demonstrated the efficacy of exosomes as delivery vehicle for microRNAs in cardiac pathologies. Exosomes from cardiac progenitor cells enriched with miR-451/144 promote cardiomyocyte survival in a myocardial ischemia/reperfusion model. To tackle the problem of cellular tropism, recently, exosomes derived from cardiosphere-derived cells (CDCs) engineered to express Lamp2b, an exosomal membrane protein, fused to a cardiomyocyte-specific peptide (CMP), resulted in an increased efficiency of exosomal uptake by cardiomyocytes (Mentkowski and Lang, 2019).

## Conclusion

The development of whole genome sequencing technologies allowed for the discovery of several classes of ncRNA and has strongly influenced our understanding of disease-regulatory circuits. LncRNAs represent one of the classes of ncRNA which has aroused more interest in the last decade. Several lncRNAs and circRNAs have been linked to cardiovascular physiology, providing new targets for the treatment of cardiovascular diseases. Additionally, the intrinsic ability of lncRNAs and circRNAs to regulate gene expression through various mechanisms makes them one of the best candidates for the development of more accurate and efficient therapies. However, important questions remain to be addressed before we can fully exploit the therapeutic potential of these molecules. Increasing our knowledge of their mechanisms of action and refining the approaches for modulating lncRNAs expression *in vivo* are two of the main challenges we still must face. Another aspect, that should be addressed, is the identification of novel lncRNAs involved in cardiovascular disease. Although the action of these RNAs is remarkably strong in the regulation of several molecular aspects, the low and time-related expression combined with a poor sequence conservation trough species makes the identification of novel lncRNAs difficult. Nevertheless, significant progress has been made in recent years, confirming the value of ncRNA as a new tool for the treatment of numerous diseases, including cardiac hypertrophy.
